# Book Reviews

**Published:** 1802-04

**Authors:** 


### CRITICAL RETROSPECT

### OF

### MEDICAL AND PHYSICAL LITERATURE.

### [ FOREIGN AND DOMESTIC. ]

*Naturhiftoriche Fragmente*; i. e. Fragments or Effays of Natural Hiftory, by G. FISCHER, Profeffor and Librarian of the Univerfity of Mentz, Vol. I. with copper-plates, pp. 253, in 4to. Frankfort, 1801. Varrentrap and Wenner.

BY the prefent publication the author purpofes to contribute materials towards improving that branch of Natural Hiftory which comprehends the internal ftructure of plants and animals, and which has by no means kept pace with what is commonly called Natural Hiftory, or with the knowledge of the external form of organic bodies. The author has, by his former publications, gained fuch credit, that we may juftly expect from his abilities and zeal the moft interefting contributions, with regard to fo extenfive and intricate a fubject.

The prefent volume contains, 1. A memoir on the great ftore of life extended over the earth, and on the attempts of Naturalifts for exploring it; read at his acceffion to the Profefforfhip of Natural Hiftory in the Univerfity of Mentz. 2. On the method in comparative parative anatomy and phyfiology; this is either comparative or monographical, that is to fay, we may contemplate an animal, or any part of it, either by itfelf in all its refpective functions, fituations, &c. or compare it with another animal, and point out the differences and their chief characteriftics. He adds here an account of the wood cuts, reprefenting fkeletons of animals, by Teodoro Filippo de Liagno; a great rarity of literature, which appeared at the beginning of the 17th century. The memoir is concluded with fome remarks on the method of reprefenting anatomical fubjects by drawing. 3. On the Mufeum of Natural History at Paris, particularly on the Cabinet for Comparative Anatomy. 4. On the different forms of the fkulls of apes, with original drawings. After having previoufly obferved, that apes, as well as makis, do not properly deferve the name of *Quadrumana*, becaufe their pofterior extremities are real feet, only with long, more moveable toes, or rather, feet with fingers, which, however, widely differ from thofe of the anterior extremities; the author proceeds to examine the fkulls of apes, particularly with refpect to the lines of face drawn by Camper, which of late has been adopted by Mr. Cuvier as the principle of divifion in the extenfive family of thofe animals. It is curious to remark, that the more the angle of face in apes approaches to that of men, the more analogy they alfo have with the human frame in other properties of organization; and, vice verfa, the more this angle recedes from that of men, the lefs fimilar they are to men in other particulars of their ftructure. For inftance, the *Orang-Outang* of *Camper* has an angle of face from 56° to 63°, which is near to that in man, whofe angle of face is 70°, and this animal alfo comes nearer to the organization of man than any other of the family of apes, viz. by the two lobes of its liver, by the proceffus vermiformis of the inteftinum cœcum, &c. This memoir contains befides, fome other remarks on the external form of the fkulls of apes, the internal ftructure of which he intends to examine in the next volume. 5. On the extraordinary minutenefs of divifion in the blood veffels of the gills of fifhes: it contains alfo fome obfervations on the liver and air-receptacles of thefe animals, as organs contributing to refpiration. 6. On the exhaling veffels of a new fpecies of *Carthamus*. 7. On foffil palms found in the umber mines of Libra near Cologne. The petrified fruits difcovered in thefe mines belong, according to Juffieu, Lamark, and Desfontaines, to a tree of the family of the palms. The firft plate reprefents the fkulls of the Orang Shimpanefe, of the Coaita and Cynophalus; the air fac of an unknown fifh called Macaibi by the Spanifh naturalifts, &c. the foffil palm fruits, &c. The fecond plate exhibits different fkulls of apes, and the fourth that of the Pongo in natural fize, after an original drawing by Camper. We cannot conclude the account of this work, without fincerely wifhing the continuance of fo interefting a publication.

---

*Lexicon nosologicum, pollyglotton, omnium morborum, symptomatum, vitiorumque naturae et affectionum propria nomina decem diversis linguis explicata continens, auctore* Ph. A. Nemnich, *Jur. Utr. Licent.* 1801, *thirty-eight sheets in folio. Hamburgh & Leipsic. Price one Louis d'or, or about* 17s.

The composition of a Scientifical Dictionary is a work attended with greater difficulties than is generally imagined, as it not only requires the knowledge of the different languages, but also of the things which make the object of it. The author happily unites both these requisites, of which his former publications are sufficient pledges. The present Dictionary contains all the generic terms of diseases in Latin, German, Dutch, Danish, Swedish, English, French, Italian, Spanish, and Portuguese, but in an inverse order, i.e. at first the Latin words with the subordinate German, English, French, &c. &c. and then the German words first, with the Latin, English, &c. explanations, and so on through all the above languages. Very few words seem to have escaped the notice of our author, who is undoubtedly entitled to our acknowledgements for this laborious and useful undertaking.

---

*C. W. Boekmann's Versuche uber das Verhalten des Phosphorus in Verschiedenen Gasarten*; i. e. Experiments on the Action of Phosphorus in different Kinds of Gases; edited by Fr. Hildebrandt, Professor of Chemistry and Medicine at the University of Erlangen, with five engravings; 1800, pp. 342, in 8vo. Erlangen for Schubart, Price 1 rixd. 12 gr. or about 5s.

The assertion of Mr. Goettling that phosphorus strongly shines in azotic gas, appeared too paradoxical to the adherents of Lavoisier's system, and too contradictory to their opinion of the nature of that gas, as not to have met with the greatest opposition on their part. The transactions, however, on this subject, seem not to have proved decisive on either side, as the antagonists of Mr. Goettling did not proceed very candidly when they asserted, that all azotic gas, in which the phosphorus happened not to shine, was pure azotic gas. The author of this publication, prepossessed by no prejudice, and merely actuated by truth and impartiality, undertook to decide the still doubtful question; in which attempt he appears to have as much succeeded as our present chemical knowledge will admit, by making for that purpose numerous experiments both with uncommon accuracy and skilfulness. A new light has been thrown on the above assertion, and at the same time that substance, so interesting to naturalists, examined in different other points of view. *Sect.* 1. *Experiments on the Action of Phosphorus in Azotic Gas.* It is extremely difficult, and almost impossible, to obtain an azotic gas which is entirely free from other substances as well as from oxygen, because the substances generally employed for separating the oxygen from the azotic gas, either leave themselves oxygen behind or are dissolved in the azotic gas; and it was owing to this circumstance that the question could not be be decided, whether phofphorus really emits light in *pure* azotic gas? Thus the azotic gas obtained by the combuftion of a fufficient quantity of phofphorus in atmofpherical air is not deftitute of oxygen, which appears from the diminution of its volume, perceived at mixing it with nitrous gas; hence the luminous appearance of phofophorus in this gas is juftly afcribed to the remaining oxygen. It may likewife be fuggefted, that when phofphorus is put into veffels, in which a fufficient quantity of it has been burned for changing the air into azotic gas, it will not fhine; but it is obvious, that the phofphoric vapours, diffolved in fuch a veffel, do here prevent its illumination. Sometimes the azotic gas is mixed with nitrous gas, which likewife obftructs the illumination. After repeated experiments the author fucceeded at laft in feparating the oxygen entirely from the atmofpherical air, by means of fulphuric alkali and the amalgama of lead, and thus to obtain a very pure azotic gas, whofe volume was not in the leaft diminifhed when brought into contact with nitrous gas in Fontana's eudiometer, and in which no fmell could be perceived. The refult of the author's obfervations is, that in fuch a pure gas the phofphorus does evidently fhine, and even ftronger than in the atmofpherical air, and, what is particularly remarkable, emits light in it at a very low temperature, viz. at 10° Reaumur, below the freezing point; whereas it ceafes to fhine in atmofpherical air at 4°—5° Reaumur above the freezing point. Phofphorus is oxydated in azotic gas and changed into phofphoric acid, and the volume of the azotic gas is diminifhed by this oxydation of phofphorus. Sometimes it happens that the illumination does not proceed in this gas long enough to perceive that diminution, which however is owing to the vapours of the phofphoric acid and oxydated phofphor that are here evolved. In order to remove thefe vapours, the author has found out a particular contrivance, but which is not to be underftood without a figure. Thefe obfervations feem to give much credit to an hypothefis, which the editor intimates in the preface, namely, that azote is perhaps compofed of oxygen and hydrogen, however inconfiftent this idea may be with our prefent theory of gafes and their refpective combinations. But a very fenfible thermometer, made for that purpofe, did not in the leaft betray any heat at this illumination of phofphorus in pure azotic gas, whereas this was immediately perceived in atmofpherical air or in azotic gas, to which a little oxygen had been previoufly added. It is, however, moft probable, that the evolution of heat in azote proceeds fo extremely flow, as not even to affect the moft fenfible inftrument.

*Sect.* 2. *Experiments on the Action of Phosphorus in Oxygen Gas.* Mr. B. made the moft accurate experiments on the abfolute confumption of oxygen gas by phofphorus being burned in it, but he never fucceeded in making it entirely difappear, whether it was prepared from the oxyd of manganefe or of mercury; there always remained a refiduum, which was indeed very little, not exceeding fometimes sometimes $\frac{1}{120}$. Phosphorus does not shine in the purest oxygen gas at low temperatures: in a temperature somewhat higher than 19°, he saw the phosphorus yield vapours, which by degrees became luminous, melted, and at last caught fire. Phosphorus, however, is oxydated in oxygen gas, even at those low temperatures in which it does not shine.

*Sect.* 3. *Action of Phosphorus in the rest of the irrespirable Gases.* In all those kinds of gases which we have hitherto become acquainted with, viz. in the carbonic and pure hydrogen gas, the carbonated, sulphurated, phosphorated hydrogen gas, in nitrous, ammoniacal, sulphurous, muriatic and fluoric gas, the phosphorus does neither fume nor shine, and even in the super-oxygenated muriatic gas, in which it is so easily inflamed, it shines but a few moments before it begins to take fire, and only in the oxydated azotic gas it fumes and shines at the same time. It is, however, to be remarked, that all these gases should be free from atmospherical air, in order to prevent the illumination of phosphorus; which, being very difficult to contrive, may account for the contradictory observations of other naturalists. All kinds of gases dissolve the phosphorus into phosphoreous vapours, which, when exposed in glass vessels to the action of sun-light, depose it again either as a dust or as crystals and vegetations of different form and colour, yellow, brown, or blackish; these are represented in the subjoined plates, together with the apparatus which the author made use of for his experiments.

---

*Dissertation sur les Fièvres pernicieuses ou ataiques intermittentes; par* J. L. Alibert, *Medecin et Membre de plusieurs Sociétés. An. viii. pp.* 139, *in 8vo. Paris, Richard, Caille, & Ravier.*

Although more than two years have elapsed since the appearance of this publication, yet we should accuse ourselves of neglect were we to omit acquainting our readers with this production of Mr. Alibert, General Secretary of the Medical Society of Emulation at Paris, whose reputation as a man of learning is sufficiently confirmed by former memoirs, scattered in sundry medical periodical publications of France. The object of this work, although we possess the excellent observations of Torti, Morton, Werthoff, Senac, and Cleghorn, is here again illustrated with new cases of irregular intermittent fevers, observed by Prof. Pinel, and by the author himself; a new classification is added, and inquiries made with respect to the aitiology of these dangerous intermittents, according to our improved knowledge of physiology and therapeutics. The classification which the author adopts, is nearly the same with that of Torti, except that he only admits varieties of this kind of fevers, whereas Torti takes them for proper species. According to this classification, there are ten varieties of the irregular intermitting fever. viz. 1. Irregular choleric or dysenteric intermittent fever. 2. Hepatic or atrabilious. 3. Cardialgical, i. e. attended with violent cardialgies and unsuccesful efforts of vomiting the contents of the the ſtomach. 4. Diaphoretic, i. e. attended with profuſe ſweats: 5. Syncopale, or attended with fainting fits. 6. Algide, i. e. freezing fever, with little ſucceeding heat. 7. Soporous. 8. Delirious. 9; which degenerates and becomes continued; and 10. Epidemic. The firſt ſeven varieties of this fever are taken from Torti, the ninth and tenth from Werthoff and Lanciſi, but the eighth has been inſerted from Mr. Alibert's own obſervations; every paroxyſm of this fever is attended with violent delirium, which comes on with the froſt. The ſecond ſection of this work is devoted to inquiries on the nature, diagnoſis, prognoſis, and treatment of theſe peculiar fevers; and in examining their proximate cauſe, the author very judiciouſly avails himſelf, according to Chauſſier, of the three principal faculties of life, that of motion, ſenſe, and the faculty of generating heat (motilité, ſenſibilité, and caloricité). He agrees with former authors in conſidering the exhalations of ſtanding water, marſhy grounds, bogs, particularly at night and during ſummer and autumn, as the remote cauſes of thoſe fevers. Bogs ſituated in high countries, chiefly when expoſed to north winds, ſeldom occaſions them. The phyſical properties of ſuch air, which is particularly apt to bring on theſe fevers, have not yet been ſufficiently examined; at leaſt, the eudiometrical obſervations, made for that purpoſe, have not proved ſatisfactory; and the author propoſes therefore a new inſtrument, which he calls hydro-eudiometer, by means of which we are likely to obtain more ſatisfactory reſults.

With reſpect to the treatment, he ſtates a ſeries of rules which we ought to follow for the cure of thoſe dangerous irregularities in the intermittent fever. He dwells particularly on the uſe of the bark, which he always recommends to give in ſubſtance, in a doſe from ſix drachms to one ounce, contrary to the common practice of French phyſicians: it ought always to be given during the apyrexy, or at leaſt at the decreaſe of the paroxyſm. We may alſo perform a cure in caſes of no imminent danger, by taking away the irregular character of the fever, and proceeding afterwards leſs vigorouſly.

---

*Geneeskundig Magazijn;* i. e. Medical Magazine, volumes firſt and ſecond, &c. 1801. Roulofswaert.

THIS Magazine or Journal is principally under the direction of Dr. Ontyd of the Hague. It is remarked, in the preface, that although Holland has long been the ſeat of Medical Knowledge, and has been rendered celebrated by the diſtinguiſhed abilities of a Boerhaave, Albinus, Camper, Van Doeveren, and many others; though it abounds with learned and able practitioners in the different branches of the healing art, yet it is deficient in that united zeal that diſtinguiſhes itſelf in other countries, and prompts the medical world to communicate occaſional obſervations and incidental diſcoveries to the public; by which both the ſcience and practice of medicine are ſo advantageouſly promoted. The Doctor, therefore, in connection with other medical gentlemen, has opened a repoſitory for that ſpecies of publication. It is not intended to publiſh this Magazine at ſtated periods, but as often as the quantity

 and and importance of the articles communicated may render it worthy of attention.

The outlines of the plan are, 1. To infert fuch difcoveries, obfervations, and experiments, which may be communicated by the eftablifhed practitioners in Holland, and fhall appear calculated to advance medical knowledge. 2. To collect the obfervations which may be made concerning the influence of different feafons of the year; the ftate of the atmofphere; fituation of towns and villages; mode of living; different occupations, &c. &c. as they affect the health of the Inhabitants. 3. To communicate propofals, plans, memoirs, &c. either originating from the Agent of the National Inftitute, or tranfmitted to him, as far as they relate to the fubject of medicine. 4. To give extracts, and make critical obfervations on medical publications, foreign and domeftic. 5. The refult of enquiries made by the National Agency, on births, marriages, deaths, &c. 6. To infert communications and brief accounts of medical writings publifhed at home or abroad; under which are comprehended, *Inaugural Dissertations, Literary Intelligence, Programmas, &c. &c.*

There is every reafon to expect that a publication of this nature will greatly contribute to the promotion of medical fcience; and we propofe to avail ourfelves of it, by inferting from time to time, fuch Extracts from it, as may prove acceptable to the Englifh reader.

